# Loss of C-Terminal Coiled-Coil Domains in SDCCAG8 Impairs Centriolar Satellites and Causes Defective Sperm Flagellum Biogenesis and Male Fertility

**DOI:** 10.3390/cells14151135

**Published:** 2025-07-23

**Authors:** Kecheng Li, Xiaoli Zhou, Wenna Liu, Yange Wang, Zilong Zhang, Houbin Zhang, Li Jiang

**Affiliations:** School of Medicine, University of Electronic Science and Technology of China, Chengdu 610072, China; likecheng_2019@hotmail.com (K.L.); zhouxiaoliyy@163.com (X.Z.); todag123@163.com (W.L.); wangyange@uestc.edu.cn (Y.W.); 13980526817@163.com (Z.Z.)

**Keywords:** SDCCAG8, centriolar satellites, male infertility, sperm flagellum biogenesis, MMAF, spermiogenesis

## Abstract

Sperm flagellum defects are tightly associated with male infertility. Centriolar satellites are small multiprotein complexes that recruit satellite proteins to the centrosome and play an essential role in sperm flagellum biogenesis, but the precise mechanisms underlying this role remain unclear. *Serologically defined colon cancer autoantigen protein 8* (*SDCCAG8*), which encodes a protein containing eight coiled-coil (CC) domains, has been associated with syndromic ciliopathies and male infertility. However, its exact role in male infertility remains undefined. Here, we used an *Sdccag8* mutant mouse carrying a CC domains 5–8 truncated mutation (c.1351–1352insG p.E451GfsX467) that models the mutation causing Senior–Løken syndrome (c.1339–1340insG p.E447GfsX463) in humans. The homozygous *Sdccag8* mutant mice exhibit male infertility characterized by multiple morphological abnormalities of the flagella (MMAF) and dysmorphic structures in the sperm manchette. A mechanistic study revealed that the SDCCAG8 protein is localized to the manchette and centrosomal region and interacts with PCM1, the scaffold protein of centriolar satellites, through its CC domains 5–7. The absence of the CC domains 5–7 in mutant spermatids destabilizes PCM1, which fails to recruit satellite components such as Bardet–Biedl syndrome 4 (BBS4) and centrosomal protein of 131 kDa (CEP131) to satellites, resulting in defective sperm flagellum biogenesis, as BBS4 and CEP131 are essential to flagellum biogenesis. In conclusion, this study reveals the central role of SDCCAG8 in maintaining centriolar satellite integrity during sperm flagellum biogenesis.

## 1. Introduction

Male infertility, affecting 25–50 million couples globally, ranks among the most prevalent reproductive disorders [1,2]. The etiology of male infertility is often ascribed to a variety of factors, including a reduced sperm count (oligozoospermia), reduced sperm motility (asthenozoospermia), increased proportion of morphologically defective spermatozoa in the ejaculate (teratozoospermia), or a combination of the above conditions [3,4]. Multiple morphological abnormalities of the sperm flagella (MMAF), a subtype of asthenoteratozoospermia, is characterized by aberrant flagellar morphologies (absent, short, bent, coiled, and irregular flagella) combined with seriously impaired sperm mobility [5]. Genetic pathogenesis is responsible for about 30–60% of MMAF cases [6]. To date, approximately 43 MMAF-associated genes have been identified that encode proteins either belonging to flagella structural components or participating in biogenesis and functions of the sperm flagellum [6,7]. Although progress has been made in exploring the genetic etiology of MMAF, the pathogenic mechanisms of MMAF remain unclear.

Centriolar satellites (hereafter satellites) are small, membrane-less, electron-dense, multiprotein complexes that enrich the area surrounding the centrosome/basal body and undergo dynamic remodeling throughout the cell cycle and in response to cell stresses [8,9,10,11,12,13]. Previous studies implied that satellites play essential roles in centrosome/basal body structural maintenance and biogenesis during centriole replication and ciliogenesis [8,12,14]. To date, over 200 satellite component proteins have been identified [11,15]. Among them, pericentriolar material 1 (PCM1) was the first satellite protein to be identified and acts as the assembly scaffold of satellites [9,14]. Depletion of PCM1 was shown to perturb the integrity of satellites and the localization and expression of other satellite components in the centrosomal region, including Bardet–Biedl syndrome 4 (BBS4), centrosomal protein of 131 kDa (CEP131), and centrosomal protein of 290 kDa (CEP290) [11,16,17,18]. Conversely, the distribution and expression of PCM1 can be disrupted by manipulation of these satellite proteins [17,18,19]. *Pcm1-null* mice exhibit syndromic ciliopathies that manifest by renal degeneration and male infertility and are characterized by sperm immobility and defects in spermiogenesis [18]. Similar sperm flagellum defects have also been observed in humans and mice carrying mutations in other satellite genes, such as *OFD1*, *BBS4*, *CEP290*, *CEP131*, and *CCDC13* [18,20,21,22,23], suggesting that satellites play a crucial role in mammalian spermiogenesis and male fertility [23]. However, the functional significance and effects of satellites on sperm flagellum biogenesis remain largely unknown.

*Serologically defined colon cancer autoantigen protein 8* (*SDCCAG8*) is classified as a ciliopathy-associated gene, and the SDCCAG8 protein localizes to centriolar satellites and the connecting cilium in the transition zone [11,24]. The human SDCCAG8 protein comprises 713 amino acids, with an N-terminal globular domain (residues 1–270), a nuclear localization signal, and eight coiled-coil domains (CC domains 1–8; residues 120–704) [25,26]. To date, 19 mutations in *SDCCAG8* have been identified in Senior–Loken syndrome (SLS) and Bardet–Biedl syndrome (BBS) patients [25,27,28,29,30,31,32,33,34,35]. Notably, all 19 mutations are nonsense or frameshift mutations leading to the C-terminal truncation of SDCCAG8. These SDCCAG8-associated patients exhibited nephronophthisis (NPHP) and retinitis pigmentosa (RP) accompanied by obesity, cognitive defects, hypogenitalism, recurrent respiratory infections, otitis media, and intracranial hypertension [25,27]. Previous in vitro studies revealed that depletion of SDCCAG8 impaired cilia formation in cultured cells [36,37]. As a highly conserved protein, *SDCCAG8* and its homologs are widely expressed in diverse species, from *Drosophila* (*CG45105*) to *Homo sapiens* (Supplementary Figure S1). Suppression of SDCCAG8 expression in zebrafish leads to defective development of the kidney, brain, and body axis [25]. Over the past few decades, six *Sdccag8* knockout and mutant mouse models have been reported. Three of them carry distinct gene-trap cassettes inserted at introns 1, 6, and 12, respectively, and have shown cilia-related abnormalities in multiple organs and systems without an analysis of male fertility, probably due to embryonic and early postnatal lethality [24,38,39]. The other three are mutant mouse models carrying different C-terminal truncated mutations (Sdccag8-Y236X, -E451GfsX467, and -R537X), with the first two generated by our group [26,40]. The three mouse models have all manifested BBS-like phenotypes, as well as male infertility. However, there are no further reports about SDCCAG8 and male infertility.

To further characterize the *SDCCAG8*-associated male infertility and to explore the underlying molecular mechanisms, in the present study, we first identified that SDCCAG8 is localized to the centrosomal region and manchette in spermatids. *Sdccag8^E451GfsX467/E451GfsX467^* mutant (hereafter *Sdccag8^mut/mut^*) male mice exhibited infertility characterized by a reduced sperm count, sperm immobility, and MMAF. Furthermore, we found that SDCCAG8 interacts with PCM1, and this interaction depends on its the fifth to seventh coiled-coil domains (CC5–7) within the C-terminal region of SDCCAG8. In the *Sdccag8^mut/mut^* spermatid, lacking this region led to the instability of PCM1 and mislocalization of the satellite-associated proteins, CEP131, and BBS4, which could be attributed to observed male infertility, as CEP131 and BBS4 are essential for flagellum biogenesis and male fertility. Those findings describe a novel role for SDCCAG8 in spermiogenesis, suggesting that mutations in *SDCCAG8* might be associated with male infertility in humans.

## 2. Materials and Methods

### 2.1. Animals

Animal experiments received approval from the Animal Care and Use Committee of Sichuan Provincial People’s Hospital (Approval No. 2014NSF(09)) and conformed to the recommendations of the Guide for the Care and Use of Laboratory Animals and institutional guidelines. Generation and genotyping protocols of *Sdccag8^mut/mut^* (*Sdccag8^E451GfsX467/E451GfsX467^*) knock-in mice were described previously [40]. All mice were maintained under 12 dark/12 light cycle conditions.

### 2.2. Reverse-Transcription PCR

The testes were collected from wild-type adult mice, and TRIzol reagent (TransGen, Beijing, China) was utilized in this total RNA extraction in accordance with the manufacturer’s guidelines. The HiScript III 1st Strand cDNA Synthesis Kit (Vazyme Biotech, Nanjing, China) was used in the cDNA synthesis progress. The primer sequences used for the analysis were as follows:

*Gapdh*-F: 5′-CGTCCCGTAGACAAAATGGT-3′;

*Gapdh*-R: 5′-TTGATGGCAACAATCTCCAC-3′;

*Sdccag8*-F: 5′-CATAGCCCTCTGGGAAGCG-3′;

*Sdccag8*-R: 5′-CTTTCAGTTGATTGACAGCATGG-3′.

### 2.3. Fertility Test

At least three sexually mature male *Sdccag8^mut/mut^* and their littermate male *Sdccag8^+/+^ Sdccag8^mut/+^* mice were individually paired with three healthy wild-type C57BL/6 female mice for three months. The date of birth and the litter size in each cage were meticulously recorded.

### 2.4. Tissue Collection and Histological Analysis

As previously reported [41], mouse testes were collected and dissected after euthanasia from at least three *Sdccag8^+/+^*, *Sdccag8^mut/+^*, and *Sdccag8^mut/mut^* mice. The testes were fixed with Bouin’s fixative solution for 24 h at 4 °C. Next, these tissues were dehydrated through graded ethanol (75%→85%→90%→95%→100%) and xylene (10 min, twice). Then, the tissues were embedded in paraffin. Sections were cut at 5 µm using the microtome (RM2016, LEICA, Wetzlar, Germany) and carefully mounted onto glass slides (G6012-1, Servicebio, Wuhan, China). The tissue sections were stained using hematoxylin and eosin (H&E) and periodic acid Schiff (PAS) according to standard procedures for further analysis.

### 2.5. Sperm Motility and Sperm Count Assays

The cauda epididymides were isolated from at least three 2-month-old *Sdccag8^+/+^*, *Sdccag8^mut/+^*, and *Sdccag8^mut/mut^* mice and shredded with tweezers. The semen samples were then released in 1 mL phosphate-buffered saline (PBS, Gibco, Grand Island, NE, USA) at 37 °C for 10 min. Then, the sperm motility was analyzed by a computer-assisted semen analysis (CASA) system (SAS Medical, Beijing, China) with a microscope through a 20× phase objective. The sperm solution was diluted at 1:300, and every 10 μL diluted sperm solution was transferred into a hemocytometer for counting. The semen analysis was repeated 4 times.

### 2.6. Transmission Electron Microscopy (TEM)

The testes dissected from *Sdccag8^+/+^*, *Sdccag8^mut/+^*, and *Sdccag8^mut/mut^* mice were fixed with 3% glutaraldehyde at 4 °C overnight. Then, samples were washed in 0.1 mol/L cacodylate buffer and postfixed with 1% OsO4 for 1 h at 4 °C, following which samples were washed with ultrapure water for 5 min. Then, the samples were dehydrated through a graded acetone series (30%→50%→70%→80%→90%→95%→100%), followed by embedding in resin (Epon-812, SPI Supplies, West Chester, PA, USA). Ultrathin sections (80 nm) were prepared using an ultramicrotome (UC7rt, LEICA, Wetzlar, Germany). The sections were transferred to a lacey formvar/carbon-film-coated copper grid (Bz11292a, Zhong Jing Ke Yi, Kaifeng, China) and stained with uranyl acetate (15 min) and lead citrate (2 min) at room temperature. Subsequently, the grid was washed with ultrapure water for 30 s. Images were acquired using a MORADA G3 camera (Emsis, Münster, Germany) mounted on a JEM-1400FLASH transmission electron microscope (JEOL Ltd., Tokyo, Japan).

### 2.7. Scanning Electron Microscopy (SEM)

As previously reported [41], spermatozoa were collected and washed twice with PBS. Then, the spermatozoa were deposited on poly-L-lysine coated slides and gently washed with phosphate-buffered saline (PBS), followed by fixation with 3% glutaraldehyde at 4 °C for 2 h. Then, samples were postfixed with 1% OsO4 for 1 h and washed with ultrapure water for 10 min. Next, the slides were dehydrated through a graded cold ethanol series (30%→50%→70%→80%→90%→95%→100%). Finally, the samples were subjected to critical point bypass drying (K850, Quorum, Laughton, UK) and gold sputtering (Smart Coater, JEOL, Tokyo, Japan). Images were acquired by a JSM-IT700HR scanning electron microscope (JEOL Ltd., Tokyo, Japan).

### 2.8. Isolation of Spermatids from Testes

Testicular spermatids’ dissociation followed established methods [42]. Tunica albuginea was peeled, and tissues were digested in DMEM containing 0.5 mg/mL collagenase IV (C4-BIOC, Sigma-Aldrich, Burlington, VT, USA) and 1.0 mg/mL DNase I (10104159001, Roche, Basel, Switzerland) at 32 °C for 30 min. Dissociated cells were centrifuged (200× *g*, 5 min), washed with PBS for three times, and fixed in 4% paraformaldehyde/PBS (15 min, room temperature). After PBS washing, cells were slide-coated and air-dried for further immunofluorescence analysis.

### 2.9. Plasmids

Full-length cDNA fragments of human *SDCCAG8* (NM.006642.5) were amplified by PCR using HEK293T cDNA as a template. The primers used to amplify *SDCCAG8* were as follows: sense and antisense primers: 5′-GCTGGACATTTCCCCACGTA-3′, 5′-GTGGTGCGTTGTGGCATATT-3′. *SDCCAG8* was subcloned into the pEGFP-N1 vector (purchased from Miaoling Biology, Wuhan, China, P0133), and then the EGFP tag was replaced by the 3× flag tag to produce a recombinant plasmid, Flag-SDCCAG8. By using the plasmid-encoding full-length SDCCAG8 as the template, the cDNA fragments encoding the human SDCCAG8-CC1-2, SDCCAG8-CC3-8, SDCCAG8-CC3-5, SDCCAG8-CC-6-8, SDCCAG8-CC4-7, SDCCAG8-CC5-8, SDCCAG8-CC5.7, SDCCAG8-CC5-6, and SDCCAG8-CC6-7 were amplified by PCR, respectively, and were subcloned into the Flag-SDCCAG8 plasmid to replace the full-length *SDCCAG8* sequence. To generate a 1339–1340insG mutation, site-directed mutagenesis (E0552S, NEB, Ipswich, MA, USA) was performed in this Flag-SDCCAG8 construct by using the following sense and antisense primers: 5′-TGGCTTCTCGGGGAAATGGAT-3′, 5′-ATCCATTTCCCCGAGAAGCCA-3′.

### 2.10. Cell Culture, Transfection, Co-Immunoprecipitation (co-IP), and MS Analysis

HEK293T cells were purchased from the National Collection of Authenticated Cell Cultures. HEK293T cells were maintained in Dulbecco’s Modified Eagle Medium (DMEM) (Gibco, Grand Island, NE, USA) with 10% FBS (Gibco, Grand Island, NE, USA) and penicillin–streptomycin (100 U/mL, Gibco, Grand Island, NE, USA) at 37 °C/5% CO_2_. Transfections used HighGene reagent (RM09014, ABclonal, Wuhan, China).

For co-IP assays, cells were harvested 48 h post-transfection and lysed with a lysis buffer (0.2% Triton-X100, 0.05 mmol/L DTT, 1× xprotease inhibitors). Then, lysates were centrifuged twice (15,000× *g*, 10 min, 4 °C). After saving 40 μL supernatant as an input, the remaining supernatant was incubated overnight at 4 °C with Anti-Flag Magnetic Beads (HY-K0207, MedChemExpress, Monmouth Junction, NJ, USA). The beads were washed 5 times with TBS buffer, and bound proteins were eluted in 50 μL 2× SDS buffer (95 °C, 5 min). Eluates were validated by Coomassie staining and Western blotting, followed by MS analysis at Oebiotech Co. (Shanghai, China).

### 2.11. Immunoprecipitation

The testicular sample preparation was performed according to the method described previously [43]. Briefly, testicular tissues of wild-type male mice were lysed with the lysis buffer. Lysates were revolved for 1 h at 4 °C and centrifuged at 13,000 rpm for 30 min. Supernatants were precleared with 30 μL of protein A/G beads (sc-2003, Santa Cruz, Dallas, TX, USA) for 3 h at 4 °C. For in vivo SDCCAG8 IP, the anti-SDCCAG8 (13471-1-AP, Proteintech, Rosemont, Chicago, IL, USA) was used. We subjected 5 μg of antibody or IgG to incubation per sample. Following overnight incubation at 4 °C, the agarose beads were washed five times with lysis buffer, and the proteins were eluted by adding 50 µL 1.5× SDS sampling buffer at 95 °C for 5 min, then analyzed by Western blotting.

### 2.12. Western Blot Analysis

Western blotting was performed following the previous protocol [44]. Mouse testes were homogenized in ice-cold protein lysis buffer (P0013K, Beyotime, Shanghai, China) containing 1× protease inhibitor cocktail (DI111-01, TransGen Biotech, Beijing, China). Protein extracts were separated by 12.5% SDS-PAGE and transferred to PVDF membranes (Millipore, Billerica, MA, USA). After blocking with 10% skim milk/TBST (2 h, room temperature), membranes were incubated with primary antibodies overnight at 4 °C. Following three TBST washes, secondary antibody incubation proceeded (2 h, room temperature). Blots were detected using an ECL–Western blotting system (Shenhua Science Technology, Hangzhou, China).

### 2.13. Immunofluorescence

Immunostaining of testis cryosections, spermatozoa, and spermatids was performed as described [45]. Briefly, sections were dried (42 °C, 15 min), washed with PBS 3 times, and blocked for 2 h at room temperature with primary blocking solution (PBS, 0.1% Triton X-100, 10% normal donkey serum, 0.02% azide). Primary antibodies diluted in blocking solution were applied and incubated overnight at 4 °C in a humidified chamber. After PBS washes, slides were incubated with secondary antibodies and DAPI (1.5 h, room temperature). Finally, images were acquired using a Zeiss LSM 900 confocal microscope (Zeiss, Baden-Württemberg, Germany).

### 2.14. Antibodies

The following primary antibodies were used: rabbit anti-SDCCAG8 (1:400 for IF, 1:2000 for WB, 1:50 for IP, 13471-1-AP, Proteintech, Rosemont, Chicago, IL, USA), mouse anti-α-tubulin (1:200 for IF, Proteintech, 66031-1-Ig), mouse anti-alpha tubulin (acetyl K40) (1:1000 for IF, ab24610, Abcam, Cambridge, UK), rabbit anti-CEP131 (1:200 for IF, 1:5000 for WB, 25735-1-AP, Proteintech), rabbit anti-BBS4 (1:500 for IF, 1:1000 for WB, 12766-1-AP, Proteintech), rabbit anti-DYKDDDDK (1:200 for IF, 1:5000 for WB, 20543-1-AP, Proteintech), and rabbit anti-PCM1 (1:300 for IF, 1:2000 for WB, 19856-1-AP, Proteintech). The Alexa Fluor 594 conjugate of lectin PNA (1:400, L32459, ThermoFisher Scientific, Carlsbad, CA, USA) was used for immunofluorescence. The following secondary antibodies were used: goat anti-rabbit Alexa Fluor 488 and 647 (1:500, Invitrogen, Waltham, MA, USA), goat anti-mouse Alexa Fluor 488 and 594 (1:500, Invitrogen), and HRP-conjugated Affinipure Goat Anti-Rabbit IgG (H+L) (1:5000, SA00001-2, Proteintech).

### 2.15. Proteomics Analysis

Fresh testis samples (four *Sdccag8^mut/+^* testes and four *Sdccag8^mut/mut^* testes) were collected and processed for proteomic analysis by Gene Denove Biotechnology Co. (Guangzhou, China) [46]. Tissues were homogenized in ice-cold lysis buffer (2% SDS, 7 M urea, 1 mg/mL protease inhibitor cocktail) using ultrasonication (3 min). Lysates were centrifuged (10,000× *g*, 15 min, 4 °C), then the supernatants were collected for protein quantification via the BCA assay. Proteins were digested with sequencing-grade trypsin (Promega; 50:1 *w*/*w*, 37 °C, 16 h). Digests underwent high-pH reverse-phase separation (Ultimate 3000 system; ThermoFisher Scientific, Carlsbad, CA, USA) using a reverse-phase column (Waters Corporation, Milford, MA, USA). Next, dried fractions were analyzed by nano-HPLC-MS/MS on an Orbitrap Fusion Lumos system coupled with EASY-nLC 1200 (Thermo Fisher Scientific, Carlsbad, CA, USA) in the data-independent acquisition (DIA) mode. Raw DIA data were processed using Spectronaut X (Biognosys AG, Zurich, Switzerland) with default parameters. Protein functional enrichment was determined by Gene Ontology (GO) analysis.

### 2.16. Conserved Motif Analysis

SDCCAG8’s amino acid sequences from human and mice were acquired from NCBI (https://www.ncbi.nlm.nih.gov/protein) (accessed on 1 May 2024). The Interpro database was employed to predict the conserved motifs in human and mouse SDCCAG8 (https://www.ebi.ac.uk/interpro/) (accessed on 1 May 2024) and those were presented and visualized by IBS (https://ibs.renlab.org/) (accessed on 1 May 2024) [47,48].

### 2.17. Protein–Protein Docking

Three-dimensional structures of SDCCAG8 and PCM1 were acquired from UniProt (AF-Q9UPN4-F1, AF-Q15154-F1). Protein–protein docking for SDCCAG8 and PCM1 was performed using AlphaFold 3 (https://golgi.sandbox.google.com/about) (accessed on 4 June 2024) [49]. The protein complex was visualized by PyMOL (Version 1.8.4.0) [50].

### 2.18. Statistical Analysis

Data are presented as mean ± SD. Statistical significance of the differences between the mean values was measured using Student’s *t*-tests. Statistical significance was defined as * *p* < 0.05, ** *p* < 0.01, *** *p* < 0.001, or **** *p* < 0.0001.

## 3. Results

### 3.1. C-Terminal Truncated Mutation of Sdccag8 Leads to Male Infertility in Mice

**Figure 1:**
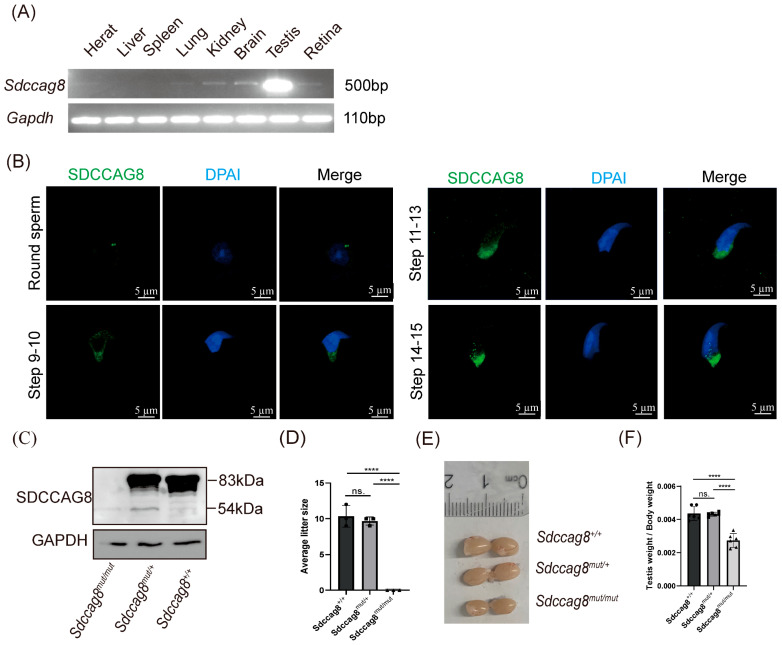
*Sdccag8* c. 1351–1352insG mutation leads to male infertility in mice. (**A**) RT-PCR analysis of *Sdccag8* mRNA expression in various tissues from wild-type C57BL/6 adult mice. (**B**) Immunofluorescence analysis of SDCCAG8 in testicular germ cells from a wild-type C57BL/6 adult mouse. Scale bars: 5 μm. (**C**) Immunoblot analysis for SDCCAG8 in protein lyases from *Sdccag8^mut/mut^*, *Sdccag8^mut/+^*, and *Sdccag8^+/+^* testes. (**D**) The average litter sizes of *Sdccag8^+/+^*, Sdccag8^mut/+^, and *Sdccag8^mut/mut^* mice at 3 months. Data are presented as means ± SD. Two-tailed Student’s *t* test; n.s., not significant; **** *p* < 0.0001. (**E**) The general appearance of the testes from *Sdccag8^+/+^*, *Sdccag8^mut/+^*, and *Sdccag8^mut/mut^*. (**F**) Quantification of the testis weight/body weight ratio in *Sdccag8^+/+^*, *Sdccag8^mut/+^*, and *Sdccag8^mut/mut^* mice (*n* = 5 mice/group). Data are presented as means ± SD. Two-tailed Student’s *t* test; n.s., not significant; **** *p* < 0.0001.

To explore the molecular functions of SDCCAG8 in spermatogenesis, we first investigated its expression and localization. Reverse-transcription PCR (RT-PCR) analysis demonstrated that *Sdccag8* was predominantly expressed in the testis, less in the heart, lung, kidney, brain, and retina, and undetectable in the liver and spleen (Figure 1A). Kamio et al. reported that SDCCAG8 is expressed in spermatocytes in the rat testis [51]. In our study, immunofluorescence (IF) analysis revealed that SDCCAG8 was detected as two spots near the nuclei of round spermatids, and it localized to the skirt-like structure encircling the spermatid head and centrosomal region from steps 9 to 15 (Figure 1B), suggesting that SDCCAG8 may be associated with spermatid elongation and flagellum biogenesis.

To determine the role of the SDCCAG8 in testicular function, we investigated *Sdccag8^mut/mut^* mice carrying a C-terminal truncated mutation (c.1351–1352insG p.E451GfsX467) that models the mutation causing Senior–Løken syndrome (c.1339–1340insG p.E447GfsX463) in humans [40]. Western blot analysis revealed that the C-terminal truncated SDCCAG8-E451GfsX467 protein (54 kDa) is expressed in the *Sdccag8^mut/mut^* mouse testes with a significantly reduced expression level compared to the full-length WT protein (83 kDa) in *Sdccag8^+/+^* and *Sdccag8^mut/+^* testes (Figure 1C). This result suggested that this C-terminal truncated mutation leads to the generation of a C-terminal truncated SDCCAG8 protein lacking CC domains 5-8 (Supplementary Figure S2). We then performed a fertility test on 3-month-old *Sdccag8^+/+^*, *Sdccag8^mut/+^*, and *Sdccag8^mut/mut^* male mice, and we confirmed that *Sdccag8^mut/mut^* males were infertile and failed to produce any offspring when mated with age-matched WT adult female mice (Figure 1D). Moreover, the testis size and the testis-to-body weight ratio in the 3-month-old mutant mice were significantly decreased compared to those of the age-matched wild-type and *Sdccag8^mut/+^* male mice (Figure 1E,F). These results confirmed that the mutation (c.1351–1352insG) in *Sdccag8* leads to male infertility with abnormal development of testes in mice.

### 3.2. Sdccag8 Mutant Males Exhibit MMAF Phenotypes

**Figure 2:**
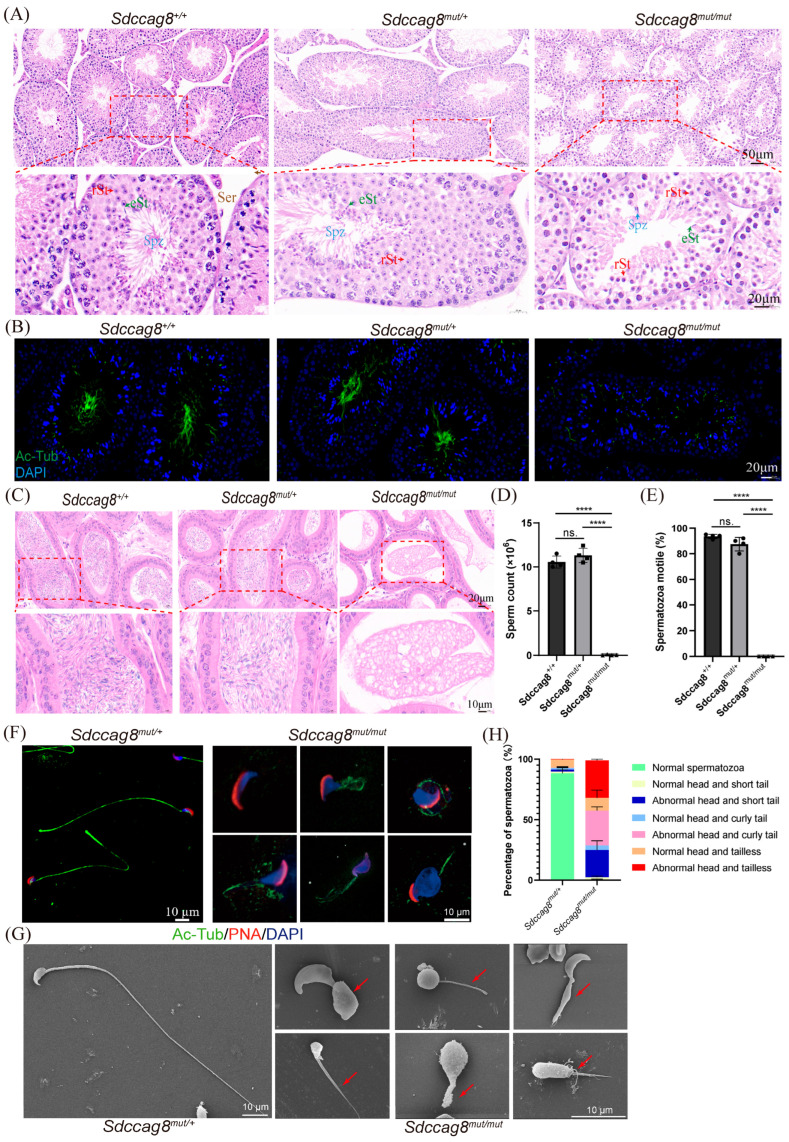
*Sdccag8* c. 1351–1352insG mutation leads to MMAF. (**A**) H&E staining of the testis sections obtained from 2-month-old *Sdccag8^+/+^*, *Sdccag8^mut/+^*, and *Sdccag8^mut/mut^* male mice. eSt, elongating spermatid; rSt, round spermatid; Spz, spermatozoa; Ser, Sertoli cells. Insets are enlarged and shown below the corresponding pictures. Scale bars: 50 μm for origin images and 20 μm for enlarged images. (**B**) Immunofluorescence analysis of Ac-Tubulin (green) and DAPI (blue) to identify the sperm flagellum in testis sections. Scale bars: 20 μm. (**C**) H&E staining of the caudal epididymis obtained from 2-month-old *Sdccag8^+/+^*, *Sdccag8^mut/+^*, and *Sdccag8^mut/mut^* male mice. Insets are enlarged and shown below the corresponding pictures. Scale bars: 20 μm for origin images and 10 μm for magnified images. (**D**) Sperm number obtained from *Sdccag8^+/+^*, *Sdccag8^mut/+^*, and *Sdccag8^mut/mut^* (*n* = 3). Data are presented as means ± SD. Two-tailed Student’s *t* test; n.s., not significant; **** *p* < 0.0001. (**E**) The percentage of motile spermatozoa from *Sdccag8^+/+^*, *Sdccag8^mut/+^*, and *Sdccag8^mut/mut^* (*n* = 3). Data are presented as means ± SD. Two-tailed Student’s *t* test; n.s., not significant; **** *p* < 0.0001. (**F**) The immunofluorescence analysis of spermatids from *Sdccag8^mut/+^* and *Sdccag8^mut/mut^* mice, the nucleus was stained with DAPI, the sperm flagella were stained with Ac-tubulin (green), the acrosome was stained with PNA lectin (red). Scale bars: 10 μm. (**G**) Scanning electron microscopy (SEM) analysis of spermatozoa from the epididymides of *Sdccag8^mut/+^* and *Sdccag8^mut/mut^* male mice. The red arrows indicate the sperm flagellum with abnormal morphology. Scale bars: 10 μm. (**H**) The percentages of different spermatozoa observed in *Sdccag8^mut/+^* and *Sdccag8^mut/mut^* caudal epididymides. Data are presented as means ± SD.

To further characterize the infertile phenotypes of *Sdccag8^mut/mut^* males in detail, we used hematoxylin and eosin (H&E) and IF staining to determine the morphology of seminiferous tubules in *Sdccag8^+/+^*, *Sdccag8^mut/mut^*, and *Sdccag8^mut/+^* mice. We found that the *Sdccag8^mut/mut^* mice have normal morphologies and numbers of spermatogonia, spermatocytes, and round spermatids, but their elongated spermatids and spermatozoa were dramatically decreased as compared with the *Sdccag8^+/+^* and *Sdccag8^mut/+^* controls at the age of 2 months (Figure 2A). In addition, there was a significant degeneration of spermatids and severely malformed flagellum of spermatozoa in the mutant testes (Figure 2A). IF analysis using anti-Ac-tub revealed severe abnormalities in the flagellar morphology of *Sdccag8^mut/mut^* spermatozoa (Figure 2B), indicating defective *Sdccag8^mut/mut^* sperm tails. We then examined the cauda epididymis of 2-month-old *Sdccag8^+/+^*, *Sdccag8^mut/+^*, and *Sdccag8^mut/mut^* male mice by H&E staining. We found fewer spermatozoa in the epididymal lumen of the *Sdccag8^mut/mut^* mice than those of the age-matched WT and HET mice (Figure 2C). After dispensing the spermatozoa from the epididymis, we found that the sperm count and mobility were dramatically reduced in *Sdccag8^mut/mut^* mice compared to WT and HET mice (Figure 2D,E). As there were no significant differences in male fertility, sperm number, and motility between HET and WT mice, we used the *Sdccag8^mut/+^* mouse as the normal control hereafter. 

To investigate the morphology of mature spermatozoa from *Sdccag8^mut/mut^* mice in detail, we analyzed epididymal spermatozoa by immunofluorescence analysis and scanning electron microscopy (SEM). The *Sdccag8^mut/mut^* caudal epididymis only contained mosaic malformations of spermatozoa exhibiting an MMAF phenotype of either a short tail, an irregular tail, a coiled tail, disordered filaments, or being tailless, with an abnormal morphology of spermatozoa heads (Figure 2F,G). The percentage of spermatozoa with abnormal heads and tails is shown in Figure 2H.

To further explore the effects of *Sdccag8* mutation on testicular protein expression, we isolated testes from four *Sdccag8^mut/mut^* mice and four *Sdccag8^mut/+^* mice at P60 for proteomics analysis. We set the threshold as a fold-change greater than 1.5 and a *p*-value less than 0.05. We quantified a total of 10,401 proteins, including 174 up-regulated and 567 down-regulated proteins (Supplementary Figure S3A). Consistent with above results in sperm flagellum anomalies, Gene Ontology (GO) enrichment analysis demonstrated that the differentially expressed proteins were associated with the motile cilium, sperm flagellum, axoneme, ciliary plasm, axonemal microtubule doublet inner sheath, axonemal microtubules, sperm middle piece, sperm principal piece, and sperm fibrous sheath (Supplementary Figure S3B). Among the 741 regulated proteins, 107 proteins were involved in the motile cilium and sperm flagellum (Supplementary Figure S3C), and all of these proteins were significantly decreased in *Sdccag8^mut/mut^* testes compared with controls, consistent with the observed morphology of the defective sperm flagellum in *Sdccag8^mut/mut^* spermatozoa.

Taken together, this *Sdccag8* mutation results in a reduced sperm count and motility, morphological abnormalities of the sperm head and flagella, as well as dysregulated sperm flagellar proteins, which collectively account for the male infertility in mice.

### 3.3. The Sdccag8 Mutation Disrupts Spermiogenesis by Causing Abnormal Manchette Formation and Nuclear Elongation

**Figure 3:**
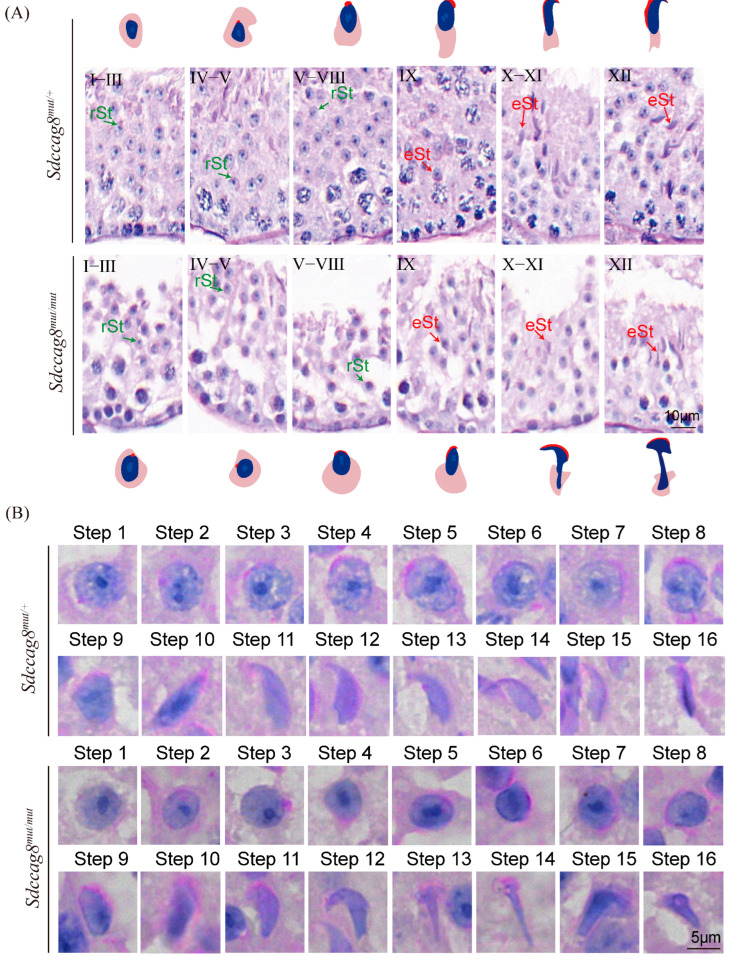
Spermiogenesis is defective in *Sdccag8^mut/mut^* mice. (**A**) PAS staining of the testis sections obtained from 2-month-old *Sdccag8^mut/+^* and *Sdccag8^mut/mut^* male mice. eSt, elongating spermatid; rSt, round spermatid. Scale bars: 10 μm. (**B**) PAS staining of spermatids at different steps from 2-month-old *Sdccag8^mut/+^* and *Sdccag8^mut/mut^* male mice, showing abnormal sperm head shape. Scale bars: 5 μm.

Spermiogenesis, which begins with the mitotic proliferation of spermatogonia in the mouse seminiferous epithelium, could be divided into 12 stages (I–XII) or 16 steps (1–16) based on sperm head and acrosome morphologies [52]. To investigate which stages of spermiogenesis are first interrupted by the *Sdccag8* mutation, we examined the morphology of spermatids across the spectrum of spermiogenesis stages by periodic acid Schiff (PAS) staining of testis sections (Figure 3A). There was no difference observed in spermatid morphologies until stage Ⅷ between 2-month-old *Sdccag8^mut/mut^* mice and the age-matched controls (Figure 3A). The morphology of the acrosome and nuclei of the *Sdccag8^mut/mut^* spermatids was as normal as that of the controls during steps 1–8 (Figure 3B). However, deformed spermatid heads and abnormally elongated sperm heads were observed in *Sdccag8^mut/mut^* testes following stage Ⅸ, and the number of their elongated spermatids was gradually reduced compared to controls (Figure 3A). Moreover, abnormal elongation of the spermatid head was observed from steps 9–16, characterized by abnormal club-shaped nuclei (Figure 3B).

**Figure 4:**
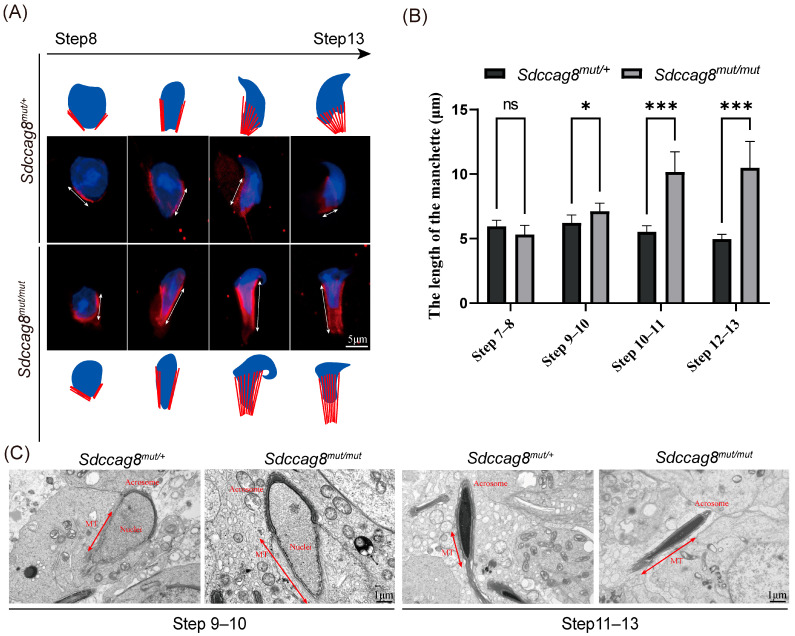
The manchette is abnormally elongated in *Sdccag8^mut/mut^* spermatids following step 9. (**A**) Immunofluorescence analysis of the manchette (double-headed arrows) in *Sdccag8^mut/+^* and *Sdccag8^mut/mut^* spermatids with anti-α-tubulin antibody (red). Nuclei were stained with DAPI (blue). The abnormal elongation of manchette in *Sdccag8^mut/mut^* spermatids could be recognized following step 9. Scale bars: 5 μm. (**B**) Statistical analysis of the length of the manchette at different steps. The length of the manchette in spermatids is *Sdccag8^mut/mut^* abnormally elongated following step 9. Data are presented as means ± SD. Two-tailed Student’s *t* test; n.s., not significant; * *p* < 0.05, *** *p* < 0.001. (**C**) Representative TEM micrographs of spermatid heads. The manchette (double-headed arrows) of elongating spermatids (steps 9–13) from *Sdccag8^mut/mut^* mice were significantly elongated, twisted, and ectopically placed with an abnormal head shape. Scale bars: 1 μm.

Spermatids undergo nuclear shaping and manchette formation following step 9 of spermiogenesis [52]. The manchette, consisting of a series of parallel microtubule bundles from the perinuclear rings of the nucleus to the distal cytoplasm, assembles concurrently with spermatid nuclear elongation and plays an essential role in sperm head shaping [53,54,55,56,57]. To investigate whether the mutation affects the elongation of the manchette in testicular spermatids during spermiogenesis, we performed IF staining and transmission electron microscopy (TEM). There were various abnormalities in manchette morphology observed in *Sdccag8^mut/mut^* spermatids, including elongated, ectopic, and disordered manchette microtubules (Figure 4A–C).Taken together, our results indicate that the *Sdccag8* mutation disrupted spermiogenesis by impairing the manchette structure and sperm head morphology following step 9 of spermiogenesis.

### 3.4. Sdccag8 c.1351–1352insG Mutation Destabilizes PCM1 and Disrupts the Centriolar Satellite Integrity in Spermatids

**Figure 5:**
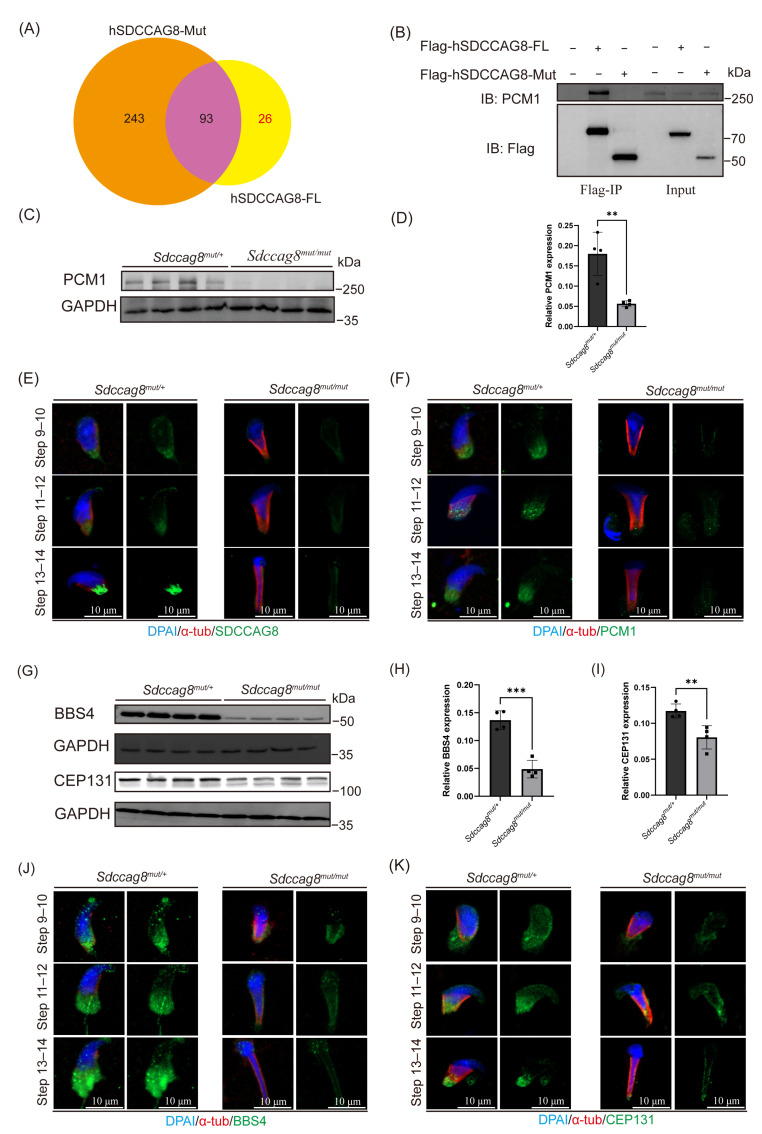
*Sdccag8* c.1351–1352insG mutation destabilizes PCM1 in spermatids and disrupts the centriolar satellites’ integrity. (**A**) Venn diagram showing overlap of proteins between the Flag-hSDCCAG8-FL binding proteins and the Flag-hSDCCAG8-Mut binding proteins. (**B**) Co-immunoprecipitation analysis of SDCCAG8 and PCM1. (**C**) Immunoblot analysis of PCM1 and SDCCAG8 in testes of *Sdccag8^mut/+^* and *Sdccag8^mut/mut^*. GAPDH was used as a loading control (*n* = 4). (**D**) Quantitative results of immunoblot. The protein level of PCM1 was normalized against GAPDH levels. Data are presented as means ± SD. Two-tailed Student’s *t* test; ** *p* < 0.01. (**E**,**F**) Representative images of spermatids obtained from *Sdccag8^mut/mut^* and *Sdccag8^mut/+^* mice testes co-stained with anti-α-tubulin, DAPI (blue), and anti-SDCCAG8 (**E**), and anti-PCM1 (**F**) (red). Scale bar: 10 µm. (**G**) Immunoblot analysis of protein expression level of BBS4 and CEP131 in protein lyases from *Sdccag8^mut/+^* and *Sdccag8^mut/mut^* testes. GAPDH served as a loading control (*n* = 4). (**H**,**I**) Quantitative results of immunoblot. Protein levels of BBS4 (**H**) and CEP131 (**I**) were normalized against GAPDH levels. Data are presented as means ± SD. Two-tailed Student’s *t* test; ** *p* < 0.01, *** *p* < 0.001. (**J**,**K**). Representative images of spermatids obtained from *Sdccag8^mut/mut^* and *Sdccag8^mut/+^* mice co-stained with anti-α-tubulin (red), DAPI (blue), and anti-BBS4 (green) (**J**), and anti-CEP131 (green) (**K**). Scale bar: 10 µm.

To investigate the molecular mechanism underlying SDCCAG8-associated flagellar defects, we attempted to obtain clues by identifying SDCCAG8-interacting proteins. To this end, we performed immunoprecipitation (IP) coupled with quantitative MS (IP-MS) using HEK293T cells stably expressing either Flag-tagged full-length human SDCCAG8 (hSDCCAG8-FL) or Flag-tagged C-terminal truncated human SDCCAG8 (hSDCCAG8-Mut; c.1339–1340insG p.E447GfsX463 in human *SDCCAG8*, corresponding to c.1351–1352insG p.E451GfsX467 in murine *Sdccag8*). We established two selection criteria to ensure the stringent identification of protein interactors: (1) peptide confidence score > 10, and (2) detection of ≥2 unique peptides per protein. Subsequent mass spectrometry analysis identified 119 potential hSDCCAG8-FL partners versus 336 potential hSDCCAG8-Mut interactors, with 26 candidate proteins showing an exclusive binding affinity for hSDCCAG8-FL (Figure 5A, Supplementary Table S1). Among these 26 proteins, PCM1 was particularly noteworthy. *Pcm1^−/−^* and *Sdccag8^mut/mut^* mice shared similar phenotypes in sperm flagellar defects and male infertility, which prompted us to investigate the potential functional association between those two proteins in flagellum biogenesis [18]. IF analysis confirmed the colocalization of SDCCAG8 and PCM1 at the centrosomal region (Supplementary Figure S4). Further co-immunoprecipitation (co-IP) and immunoblotting analyses demonstrated that SDCCAG8 interacted with PCM1 in HEK293T cells and WT mouse testicular extract (Figure 5B, Supplementary Figure S5A), and that interaction was disrupted by this C-terminal truncated mutation (Figure 5B).

To determine the impact of this *Sdccag8* mutation on the expression of PCM1 in vivo, Western blot analysis was conducted. A significantly reduced expression level of PCM1 was identified in *Sdccag8^mut/mut^* testes (Figure 5C,D). Subsequent IF analysis indicated that PCM1 and SDCCAG8 were localized at the manchette and centrosomal region in HET control spermatids. In contrast, the fluorescence intensities of SDCCAG8 and PCM1 were significantly reduced in *Sdccag8^mut/mut^* spermatids (Figure 5E,F). These results suggest that SDCCAG8 may regulate the stability of PCM1 in spermatids.

Previous studies have demonstrated that PCM1 deficiency not only disrupted the centriolar satellite integrity but also affected the expression levels of satellite components [9,14,58]. In light of the observed reduction in PCM1 in *Sdccag8^mut/mu^^t^* spermatids, we speculated that the satellite integrity might be compromised. To evaluate this speculation, we first analyzed the above proteomics data and found that 41 centriolar satellite proteins were significantly altered in *Sdccag8^mut/mu^*^t^ testes, with 11 up-regulated and 30 down-regulated (Supplementary Figure S5B). Among these 41 proteins, we focused on BBS4 and CEP131, two PCM1-interacting satellite components, due to their essential roles in flagellum biogenesis and male fertility in mice [18,20,21]. Subsequent validations of the reductions in both proteins in *Sdccag8^mut/mu^^t^* testes by Western blotting were consistent with the findings in proteomic data (Figure 5G–I). In *Sdccag8^mut/+^* spermatids, BBS4 was present in the centrosomal region and the elongating sperm tails, while CEP131 was enriched at the centrosomal region as spermatids developed. However, we found that BBS4 and CEP131 both lost their centrosomal location and mislocalized to the manchette in *Sdccag8^mut/mut^* spermatids (Figure 5J,K), suggesting that SDCCAG8 is required for the proper localization of BBS4 and CEP131. Taken together, these results suggest that this C-terminal truncated mutation in *Sdccag8* disrupts its interaction with PCM1, destabilizes PCM1, and impairs the structural integrity of satellites, which likely constitutes the molecular basis for observed flagellar defects.

### 3.5. SDCCAG8 Interacted with PCM1 Through Its Fifth to Eighth Coiled-Coil Domains

**Figure 6:**
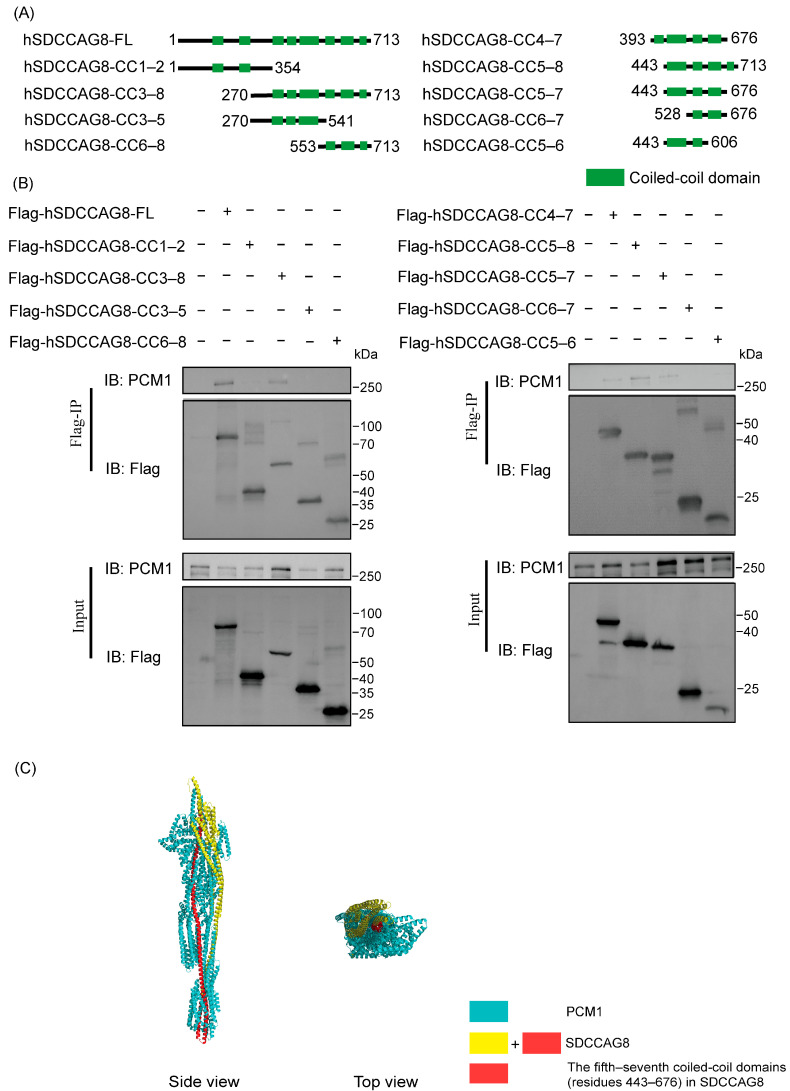
The region consisting of the fifth to the seventh coiled-coil domains in SDCCAG8 (residues 443–676) is essential for the interaction with PCM1. (**A**) Schematic diagrams of human SDCCAG8-FL, -CC1–2, -CC3–8, -CC3–5, -CC6–8, -CC4–7, -CC5–8, -CC5–7, -CC6–7, -CC7–8. (**B**) Co-immunoprecipitation analysis of hSDCCAG8 and endogenous PCM1. (**C**) Binding model of SDCCAG8 and PCM1 was constructed by AlphaFold 3 (https://golgi.sandbox.google.com/about) (accessed on 4 June 2024). The protein complex is visualized by PyMOL. The region of the fifth–seventh coiled-coil domains (residues 443–676) marked by red located in the core area of this protein–protein complex.

A previous study revealed there were eight coiled-coil domains in hSDCCAG8 (Figure 6A). To further identify which C-terminal CCs of hSDCCAG8 play a pivotal role in its interaction with PCM1, we performed a co-immunoprecipitation analysis of endogenous PCM1 with flag-tagged full-length hSDCCAG8 protein, hSDCCAG8-FL, and four distinct truncated mutants, hSDCCAG8-CC1–2, -CC3–8, -CC3–5, and -CC6–8, stably expressed in cultured HEK293T cells (Figure 6A). Among the five proteins, only the hSDCCAG8-FL and hSDCCAG8-CC3–8 co-precipitated with PCM1 (Figure 6B). To further narrow down the PCM1-interacting domains within hSDCCAG8, we then generated a series of truncated hSDCCAG8 mutants, hSDCCAG8-CC4–7, -CC5–8, -CC5–7, -CC6–7, and -CC5–6. Among them, hSDCCAG8-CC4–7, -CC5–8, and -CC5–7 co-precipitated with PCM1, whereas hSDCCAG8-CC6–7 and -CC5–6 did not, indicating that CC5–7 is essential for SDCCAG8-PCM1 interaction (Figure 6B). To gain further insight into the role of CC5–7 in the binding of SDCCAG8 to PCM1, a structural model of the hSDCCAG8-PCM1 complex was constructed by AlphaFold3 [49]. The model positioned CC5–7 at the core of the predicted club-shaped complex, suggesting its essential role in this interaction (Figure 6C).

To date, 19 *SDCCAG8* mutations have been identified in patients [25,27,28,29,30,31,32,33,34,35]. Of note, these mutations were nonsense or frameshift mutations distributed from exon 5 to exon 16, leading to a truncation of the C-terminal domain of SDCCAG8. Importantly, all 19 mutations lie within or upstream of this region required for SDCCAG8-PCM1 interaction (Supplementary Figure S6), underscoring the critical function of this region.

## 4. Discussion

Since its initial characterization in 2014 [59], the MMAF phenotype has been characterized by sperm tail abnormalities, including short, coiled, absent, or irregular flagella; however, its genetic underpinnings remain unclear [6]. This study is the first to elucidate the function of SDCCAG8 in spermiogenesis. SDCCAG8 is located at the manchette and centrosomal region of spermatids. A C-terminal truncated mutation (c.1351–1352insG) in *Sdccag8* led to male infertility and a typical MMAF phenotype in male mice with defects in sperm head shaping and manchette formation. In an immunoprecipitation study, we found that SDCCAG8 interacts with PCM1, the scaffold of centriolar satellites. This C-terminal truncated mutation disrupted the interaction between SDCCAG8 and PCM1, leading to the significantly decreased expression of PCM1 and the disruption of satellite integrity in spermatids. Collectively, our results indicate that the C-terminal region of SDCCAG8 plays an essential role in flagellum biogenesis and male fertility in mice.

Protein trafficking to specific cellular regions is crucial for ciliogenesis and sperm flagellum biogenesis [60]. The centrosome serves as the template for cilia assembly, where ciliary and centrosomal proteins are enriched [61,62,63]. Satellites are the small membraneless multi-protein complexes enriched in the centrosomal region, serving as a platform to recruit satellite proteins to the centrosomal region along microtubules [10,11,14,18]. Emerging evidence implies the pathological involvement of satellites in sperm flagellum defects and male infertility. Several satellite genes have been associated with male infertility and sperm flagellum defects in humans and mice, including *PCM1*, *CEP131*, *BBS4*, *OFD1*, *CEP290*, *CCDC13*, etc. [18,20,21,22,23]. However, further investigation is required to determine whether additional satellite-related genes are involved in sperm flagellum formation. SDCCAG8 was identified as a syndromic ciliopathy-associated gene in 2010, and was further proven to be a satellite protein due to its colocalization and interaction with PCM1 [24,25]. Consistent with the findings of other satellite genes in the sperm flagellum, our study showed that *Sdccag8^mut/mut^* mice exhibited flagellum defects and male infertility, emphasizing the vital roles of satellites in sperm flagellum biogenesis and male fertility.

PCM1 functions as a scaffolding protein essential for the satellite assembly [9,19,64]. Deletion of PCM1 disrupts the integrity of satellites and further affects satellite protein localization and expression, pericentriolar material protein enrichment, microtubule organization, and ciliogenesis [8,10,12,14]. In 2014, Ryan et al. reported that SDCCAG8 interacts, colocalizes, and co-traffics with PCM1 in COS7 cells [24]. Further mass spectrometry-based proteome profiling and proximity-dependent biotin identification studies further confirm the PCM1-SDCCAG8 interaction within centriolar satellites [11,16]. Dysfunction of SDCCAG8 in RPE1 and COS7 cells disrupts pericentriolar material accumulation, microtubule organization, and ciliogenesis [24,37]. *Pcm1-null* and *Sdccag8^mut/mut^* mice share similar phenotypes in syndromic ciliopathy and male infertility, including renal degeneration, perinatal lethality, a reduced sperm number, sperm immobility, an abnormal head morphology and spermiogenesis defects [18]. These results suggest the potential functional association between SDCCAG8 and PCM1. In this study, we confirmed the interaction between SDCCAG8 and PCM1, and we further found a significantly decreased PCM1 expression in *Sdccag8^mut/mut^* spermatids. Further investigations are necessary to elucidate the detailed roles of SDCCAG8 in PCM1 stability and their relationships during flagellum biogenesis.

Previous studies have revealed that the location pattern and expression level of BBS4 and CEP131 depend on satellites, while dysfunction of satellites impairs BBS4 and CEP131 localization to the centrosomal region [17,58]. Importantly, BBS4 and CEP131 play an essential role in flagellum biogenesis. BBS4 is an adapter for the transport of ciliary membrane proteins to the cilia via the intra-flagellar transport (IFT) system [65,66]. *Bbs4-null* mice exhibited obesity, retinal degeneration, male infertility, and flagellum defects [20,67]. CEP131 is a highly conserved and ubiquitously expressed coiled-coil protein and plays an essential role in cilia formation in flies and zebrafish [68,69]. *Cep131-null* mice exhibited male infertility and sperm flagella defects [21,70]. In this study, we found that BBS4 and CEP131 lose their location to the centrosomal region with reduced expression levels in *Sdccag8^mut/mut^* spermatids. We attribute these alterations to the dysfunction of satellites, which could further disrupt sperm flagellar biogenesis in *Sdccag8^mut/mut^* mice.

Satellite dysfunction is associated with the disorganized manchette. The manchette, a transient microtubule and actin-based structure, plays a role in nuclear remodeling and protein transport to sperm heads and tails [54,71]. Distinct from the axonemal microtubules, manchette microtubules are oriented with their plus-ends toward the acrosome and minus-ends extend toward the caudal side [54,72,73,74]. How the manchette is formed is still not clear. Manchette microtubules consist of head-to-tail heterodimers of α- and β-tubulin. γ-Tubulin is not a part of microtubules’ polymer, but forms the γ-tubulin ring complex, which serves as the template for microtubule nucleation and regulates the stabilization of the minus-ends of manchette microtubules [75,76]. Previous studies of the satellite proteome and interactome have revealed the diverse functions of satellites, including the organization of microtubules and the nucleation of actin [11,77]. Dysfunction of SDCCAG8 or PCM1 impairs the centrosomal accumulation of γ-tubulin in COS7 cells and mouse neurons [24]. Importantly, *Pcm1^−/−^* and *Sdccag8^mut/mut^* mice share a similar defect in the manchette structure with the abnormally elongated spermatid heads [18,21]. Collectively, we find that dysfunction of SDCCAG8 impairs the organizations of the manchette, suggesting satellites’ potential role in the manchette microtubule organization during spermiogenesis. Of note, we found that SDCCAG8 interacts with CEP170 and KIF2a (Supplementary Table S1). CEP170 and KIF2a could form a complex that is associated with the manchette integrity and dynamics. Dysfunction of this complex leads to abnormal elongation of the manchette [72].

In agreement with previous reports that the C-terminal region of SDCCAG8 plays an essential role in primary cilia formation, our findings also reveal the importance of the C-terminal region of SDCCAG8 in the sperm flagella biogenesis. The human *SDCCAG8* genotypes identified in patients are all biallelic deletion, insertion, nonsense, or splicing variants distributed across exons 5 to 16, resulting in the production of C-terminal truncated proteins of variable size [40]. The C-terminal truncated mutation in humans and mice leads to similar syndromic ciliopathy phenotypes, and the severity of the phenotypes was negatively proportional to the hypomorphic strength of the *Sdccag8* mutations, Y236X and E451GfsX467 [40]. Previous studies have reported that the C-terminal region of SDCCAG8 regulates its stability and localization to the centrosome [26,40]. Additionally, the C-terminal region also serves as a module essential for its interactions with several ciliary proteins, such as RABEP2 and OFD1 [25,26]. In this study, we identified a specific region consisting of CC domains 5–7 at the C-terminal region of SDCCAG8 as essential for SDCCAG8-PCM1 interaction as well as PCM1 stability. Importantly, all known *SDCCAG8* mutations could disrupt the integrity of this region (Supplementary Figure S6). Therefore, we hypothesize that CC5–7 may constitute a functional module of SDCCAG8, and mutations that disrupt the integrity of CC5–7 may contribute to male infertility and flagellum defects in humans. This needs to be verified in future clinical studies.

Collectively, this work establishes SDCCAG8 as a critical regulator of flagellum biogenesis through its partnership with PCM1 in maintaining centriolar satellite integrity. Our findings propose a novel paradigm in which satellite dysfunction underlies specific forms of MMAF, expanding the genetic spectrum of male infertility disorders with potential implications for disease diagnosis and intervention.

## 5. Conclusions

This study elucidates the critical role of SDCCAG8 in sperm flagellum biogenesis through its regulation of centriolar satellite integrity. By characterizing an *Sdccag8* mutant mouse model carrying a truncation (c.1351–1352insG), we demonstrate that loss of SDCCAG8’s C-terminal CC domains 5–8 causes severe male infertility characterized by MMAF phenotypes, aberrant manchette organization, and defective nuclear elongation. Mechanistically, we establish that CC domains 5–7 are essential for SDCCAG8 to interact with PCM1, the scaffolding hub of centriolar satellites. Disruption of this interaction destabilizes PCM1 and impairs the recruitment of key satellite components (BBS4 and CEP131) to the centrosomal region in spermatids. This cascade ultimately compromises flagellar formation.

Notably, our findings bridge human genetics with functional validation: the mutation in mice (equivalent to human c.1339–1340insG in Senior–Løken syndrome patients) directly links the C-terminal truncated SDCCAG8 to defects in flagellum biogenesis. The evolutionary conservation of SDCCAG8 across mammals underscores its essential role in fertility.

These results position SDCCAG8 as a master regulator of centriolar satellite dynamics during sperm flagellum assembly. From a translational perspective, mutations in SDCCAG8 disrupting CC domains 5–7 should be prioritized in genetic screening for idiopathic MMAF patients. Future studies should focus on whether satellite-targeted interventions can rescue flagellar defects in SDCCAG8-deficient models, offering potential therapeutic avenues for ciliopathy-associated infertility.

While our study establishes SDCCAG8 as a key regulator of centriolar satellite integrity in sperm flagellum biogenesis, the major limitation is the lack of clinical evidence. While male infertility was observed exclusively in the *Sdccag8* mutant mice in this study, and the phenotypes are highly similar between mice and humans carrying the same mutation, direct evidence linking *SDCCAG8* mutations to human MMAF is absent. Future studies are required to screen idiopathic MMAF patients for *SDCCAG8* mutations, especially the mutations that disrupt the integrity of CC5–7, and correlate those with the PCM1/CEP131/BBS4 localization pattern in spermatozoa.

## Data Availability

The data presented in this study are available upon request from the corresponding author.

## Supplementary Materials

The following supporting information can be downloaded at https://www.mdpi.com/article/10.3390/cells14151135/s1; Figure S1: Phylogenetic tree of the SDCCAG8 homologous proteins in different species; Figure S2: The SDCCAG8 c.1339–1340insG mutation in humans and c.1351–1352insG mutation in mice result in the deletion of the fifth to eighth coiled-coil domains; Figure S3: The proteomics analysis identifies the dysregulated flagellar proteins in Sdccag8mut/mut testes; Figure S4: SDCCAG8 and PCM1 colocalized to the proximal end of primary cilia in 293T cell, and satellite proteins showed dysregulated expression levels in Sdccag8mut/mut mice testes; Figure S5: Co-immunoprecipitation experiment reveals the interaction between SDCCAG8 and PCM1 in testicular lysates; Figure S6: All known pathogenic mutations in SDCCAG8 are distributed within or upstream of the region consisting of CC domains 5–7; Table S1: The specific SDCCAG8-interacting proteins identified by co-immunoprecipitation coupled with mass spectrometry.

## Abbreviations

The following abbreviations are used in this manuscript:

BBS	Bardet–Biedl syndrome
CCs	Coiled-coil domains
H&E	Hematoxylin and eosin
IF	Immunofluorescence
IFT	Intra-flagellar transport
IP	Immunoprecipitation
MMAF	Multiple morphological abnormalities of the flagella
NPHP	Nephronophthisis
PAS	Periodic acid Schiff staining
RP	Retinitis pigmentosa
SDCCAG8	Serologically defined colon cancer autoantigen protein 8
SEM	Scanning electron microscopy
SLS	Senior–Løken syndrome
TEM	Transmission electron microscopy
