# Supplementary material for: Loss of C-Terminal Coiled-Coil Domains in SDCCAG8 Impairs Centriolar Satellites and Causes Defective Sperm Flagellum Biogenesis and Male Fertility
Source: Cells. 2025 Jul 23;14(15):1135. doi: 10.3390/cells14151135 (PMC12346347; doi:10.3390/cells14151135)
Supplement: Supplementary file 1 [file cells-14-01135-s001.zip › cells-3735898-supplementary.pdf]

Supporting Information

Loss of C-Terminal Coiled-Coil Domains in SDCCAG8 Impairs Centriolar Satellites and Causes Defective Sperm Flagellum Biogenesis and Male Fertility

Kecheng Li, Xiaoli Zhou, Wenna Liu, Yange Wang, Zilong Zhang, Houbin Zhang * and Li Jiang *

School of Medicine, University of Electronic Science and Technology of China, Chengdu 610072, China; likecheng_2019@hotmail.com (K.L.); zhouxiaoliyy@163.com (X.Z.); todag123@163.com (W.L.); wangyange@uestc.edu.cn (Y.W.); 13980526817@163.com (Z.Z.)
* Correspondence: houbin_zhang@yahoo.com (H.Z.); u0341992@yahoo.com (L.J.).

Supplementary Figure S1. Phylogenetic tree of the SDCCAG8 homologous proteins in different species.

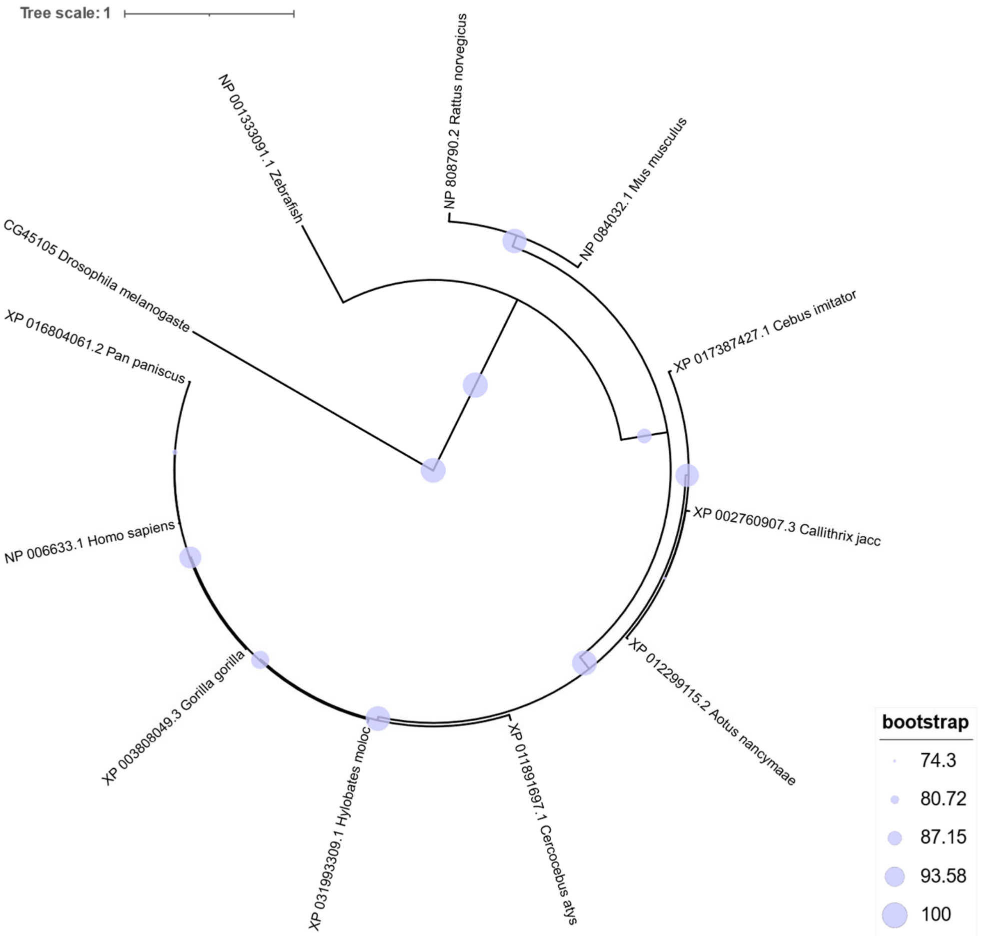

Phylogenetic tree analysis reveals the conservations of SDCCAG8 protein sequences among different species from *Drosophila melanogaster* to *Homo Sapiens*. The tree was generated using MEGA, and visualized with iTOL (https://itol.embl.de/) (accessed on 1 July, 2025).

Supplementary Figure S2. The *SDCCAG8* c.1339-1340insG mutation in humans and c.1351-1352insG mutation in mice result in the deletion of the fifth to eighth coiled-coil domains.

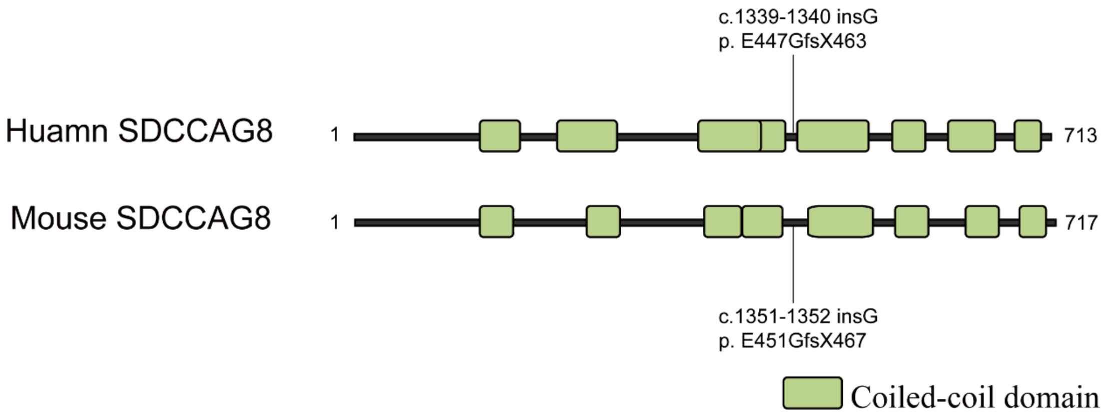

Human SDCCAG8 contains eight conserved coiled-coil (CC) domains: CC1 (residues 130–171), CC2 (220–271), CC3 (355–393), CC4 (408–442), CC5 (456–529), CC6 (550–584), CC7 (608–656), and CC8 (677–704). The human *SDCCAG8* c.1339–1340insG mutation (equivalent to murine *Sdccag8* c.1351–1352insG) generates a C-terminal truncated SDCCAG8 protein lacking CC5–8 domains.

Supplementary Figure S3. The proteomics analysis identifies the dysregulated flagellar proteins in *Sdccag8*$^{mut/mut}$ testes.

(A)

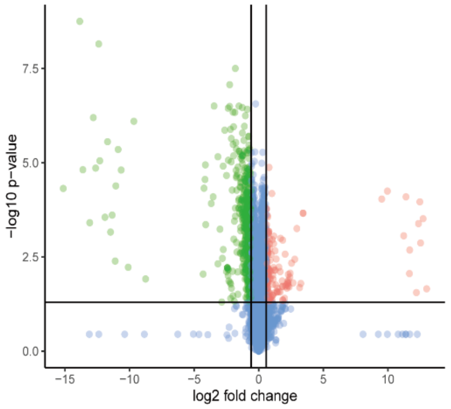

(B)

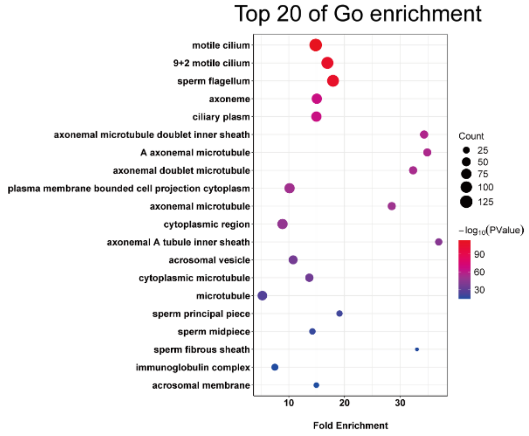

(C)

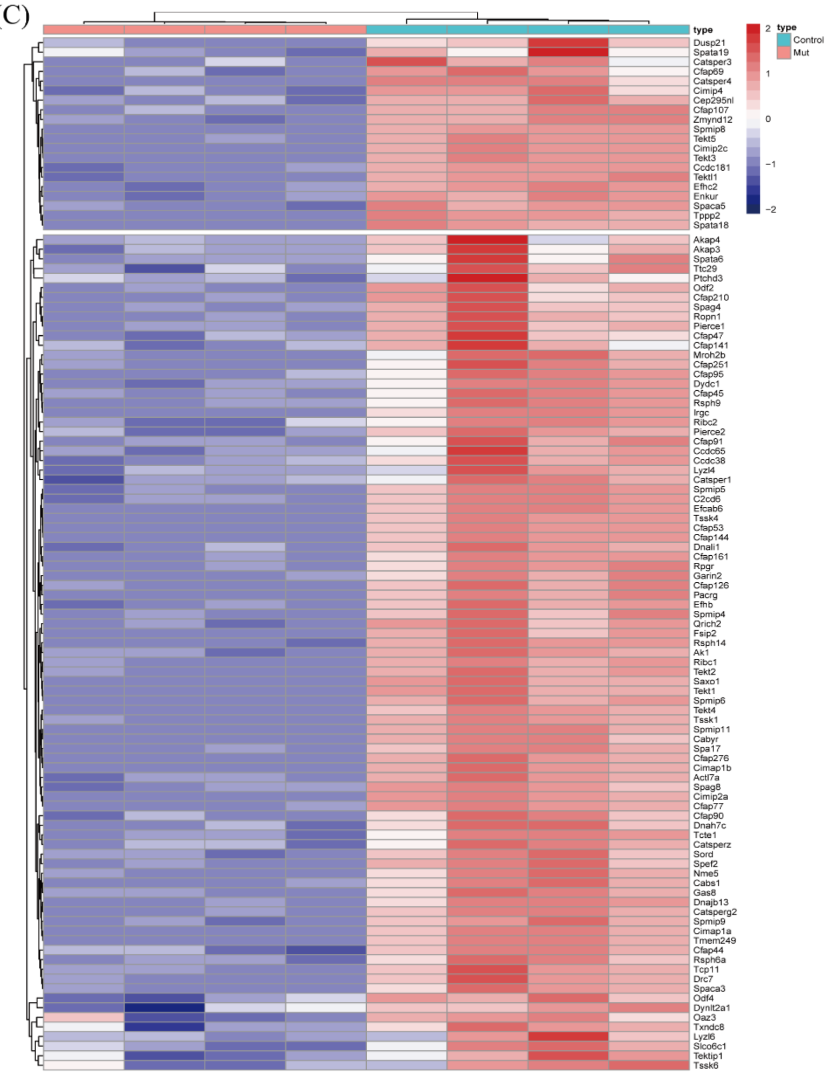

A. Volcano plot showing the differently expressed proteins (DEPs) between *Sdccag8*$^{mut/mut}$ and *Sdccag8*$^{mut/+}$ testes. Downregulated proteins are presented by green dots, upregulated proteins are represented by red dots, and proteins with no significant differential expression are represented by blue dots. We set the threshold as a fold-change greater than 1.5 and a p-value less than 0.05.

B. Bubble chart showing the enrichment of DEPs according to the GO analysis. These enriched proteins were

mainly involved in cilium, motile cilium, sperm flagellum, axoneme, ciliary plasm, axonemal microtubule doublet inner sheath, axonemal microtubules, sperm middle piece, sperm principal piece, sperm fibrous sheath et al (arranged by q-value).

C. Heatmap showing DEPs involved in motile cilium as well as flagellum in the testes of *Sdccag8*$^{mut/mut}$ mice and *Sdccag8*$^{mut/+}$ mice. All these 107 proteins were significantly decreased in *Sdccag8*$^{mut/mut}$ testes compared to *Sdccag8*$^{mut/+}$ testes.

Supplementary Figure S4. SDCCAG8 and PCM1 colocalized to the proximal end of primary cilia in 293T cell, and satellite proteins showed dysregulated expression levels in *Sdccag8*$^{mut/mut}$ mice testes..

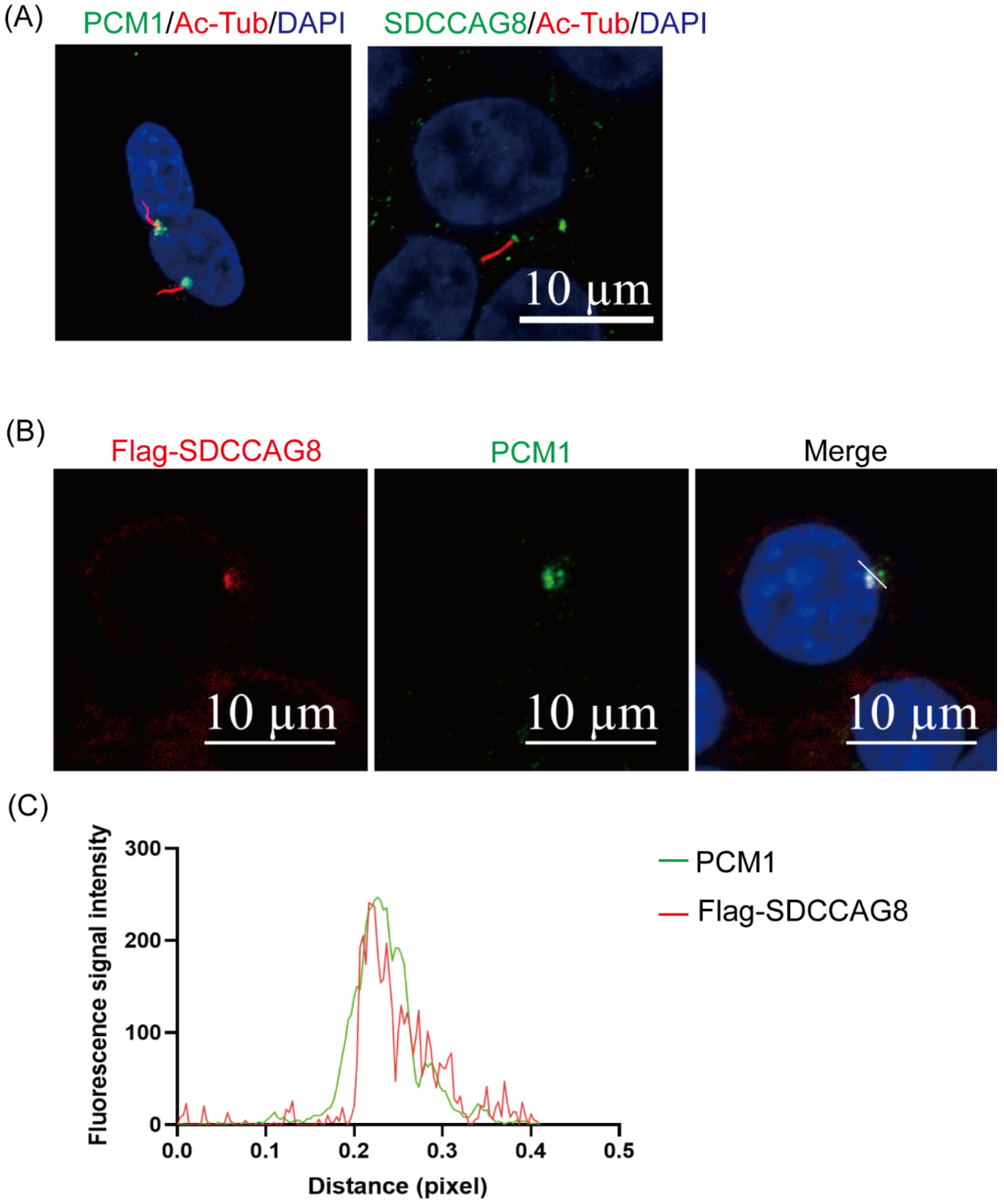

A. Immunofluorescent analysis of HEK293T cells using anti-SDCCAG8, anti-PCM1, and anti-acetylated α-tubulin antibodies (green). Nuclei were stained with DAPI (blue). Scale bars: 10 μm.

B-C. Plasmid expressing a Flag-tagged SDCCAG8 was transfected into HEK293T cells followed by immunostaining with anti-FLAG, anti-PCM1, and anti-acetylated α-tubulin antibodies (green) (B). Nuclei were stained with DAPI. Fluorescence intensity traces (offset white line) are plotted blow (C) . Scale bars: 10 μm.

Supplementary Figure S5. Co-immunoprecipitation experiment reveals the interaction between SDCCAG8 and PCM1 in testicular lysates

(A)

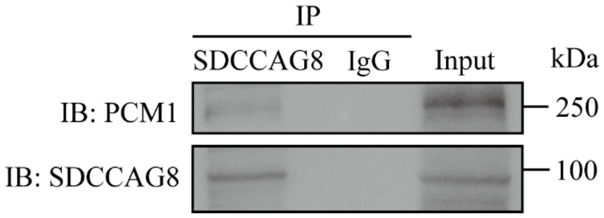

(B)

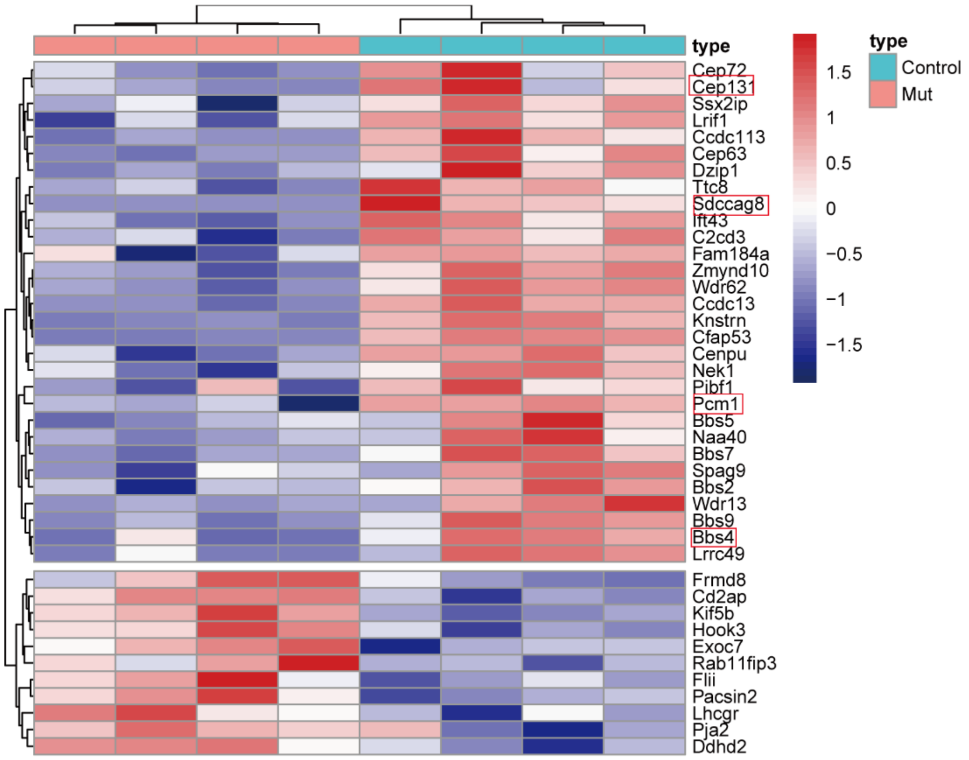

A. Co-IP assays to validate the interaction between SDCCAG8 and PCM1 in the testis. Anti-rabbit IgG was used as a control.

Heatmap showing the differentially expressed satellites-associated proteins between *Sdccag8*$^{mut/mut}$ and *Sdccag8*$^{mut/+}$ mice testes. There were 41 differentially expressed centriolar satellite proteins in *Sdccag8*$^{mut/mut}$ mutants, with 11 upregulated and 30 downregulated. PCM1, SDCCAG8, CEP131, BBS4 showed a significant reduced expression levels in *Sdccag8*$^{mut/mut}$ testes.

Supplementary Figure S6. All known pathogenic mutations in *SDCCAG8* are distributed within or up-stream of the region consisting of CC domains 5–7.

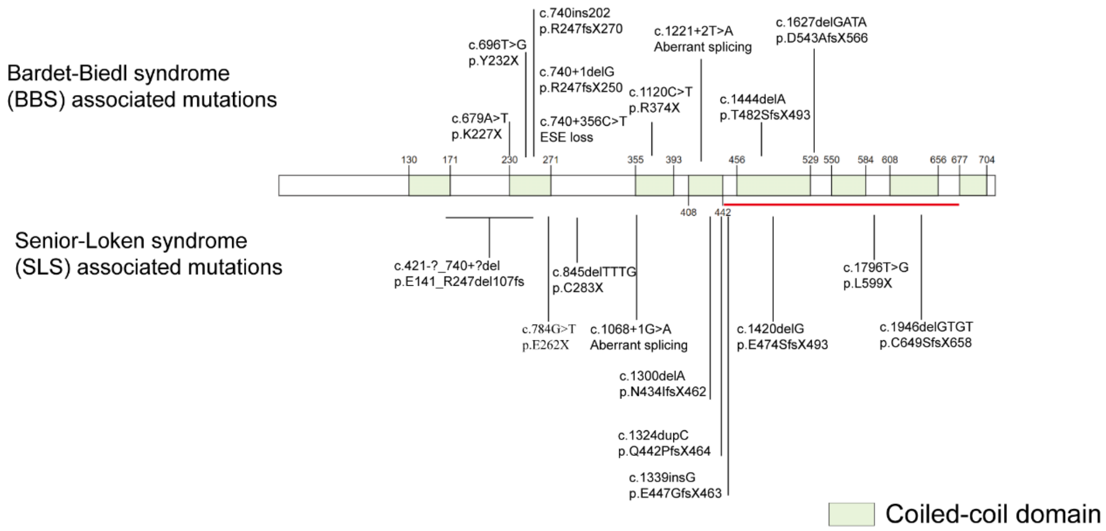

Schematic representation of the SDCCAG8 protein. The CC domains **5**–**7** were highlighted by a red line. This diagram illustrates all 19 mutations identified in patients to date. Notably, all 19 mutations result in the truncation of the C-terminal region of SDCCAG8. Each of these mutations is located within or upstream of CC**5**–**7** (red line).

Supplementary Table S1. The specific SDCCAG8-interacting proteins identified by co-immunoprecipitation coupled with mass spectrometry.

Gene Symbol	Full name	No. unique peptides	Score
CEP170	Centrosomal protein of 170 kDa OS=Homo sapiens OX=9606 GN=CEP170 PE=1 SV=1	47	239.126
KIF2A	Kinesin-like protein KIF2A OS=Homo sapiens OX=9606 GN=KIF2A PE=1 SV=3	25	125.321
TUBA1A	Tubulin alpha-1A chain OS=Homo sapiens OX=9606 GN=TUBA1A PE=1 SV=1	13	65.182
CPNE3	Copine-3 OS=Homo sapiens OX=9606 GN=CPNE3 PE=1 SV=1	9	47.403
EEF1A1	Elongation factor 1-alpha 1 OS=Homo sapiens OX=9606 GN=EEF1A1 PE=1 SV=1	8	46.54
JUP	Junction plakoglobin OS=Homo sapiens OX=9606 GN=JUP PE=1 SV=3	7	31.236
ANXA2	Annexin A2 OS=Homo sapiens OX=9606 GN=ANXA2 PE=1 SV=2	7	21.074
SERPINB3	Serpin B3 OS=Homo sapiens OX=9606 GN=SERPINB3 PE=1 SV=2	7	20.477
CSTA	Cystatin-A OS=Homo sapiens OX=9606 GN=CSTA PE=1 SV=1	6	22.818
PCM1	Pericentriolar material 1 protein OS=Homo sapiens OX=9606 GN=PCM1 PE=1 SV=6	4	16.85
GNAS	Guanine nucleotide-binding protein G(s) subunit alpha isoforms XLas OS=Homo sapiens OX=9606 GN=GNAS PE=1 SV=2	5	11.331
DCD	Dermcidin OS=Homo sapiens OX=9606 GN=DCD PE=1 SV=2	4	22.596
AIMP2	Aminoacyl tRNA synthase complex-interacting multifunctional protein 2 OS=Homo sapiens OX=9606 GN=AIMP2 PE=1 SV=2	4	17.039
S100A8	Protein S100-A8 OS=Homo sapiens OX=9606 GN=S100A8 PE=1 SV=1	4	16.114
GNB1	Guanine nucleotide-binding protein G(I)/G(S)/G(T) subunit beta-1 OS=Homo sapiens OX=9606 GN=GNB1 PE=1 SV=3	4	15.882
HP	Haptoglobin OS=Homo sapiens OX=9606 GN=HP PE=1 SV=1	4	14.388
FLG	Filaggrin OS=Homo sapiens OX=9606 GN=FLG PE=1 SV=3	4	11.004
MAP4K4	Mitogen-activated protein kinase kinase kinase kinase 4 OS=Homo sapiens OX=9606 GN=MAP4K4 PE=1 SV=2	4	10.131
GNAI3	Guanine nucleotide-binding protein G(i) subunit alpha-3 OS=Homo sapiens OX=9606 GN=GNAI3 PE=1 SV=3	3	46.54
IGHG2	Immunoglobulin heavy constant gamma 2 OS=Homo sapiens OX=9606 GN=IGHG2 PE=1 SV=3	3	14.504
HNRNPA1L3	Heterogeneous nuclear ribonucleoprotein A1-like 3 OS=Homo sapiens OX=9606 GN=HNRNPA1L3 PE=4 SV=2	3	12.297
S100A9	Protein S100-A9 OS=Homo sapiens OX=9606 GN=S100A9 PE=1 SV=1	3	16.287
SERPINB12	Serpin B12 OS=Homo sapiens OX=9606 GN=SERPINB12 PE=1 SV=1	3	11.541
RPA1	Replication protein A 70 kDa DNA-binding subunit OS=Homo sapiens OX=9606 GN=RPA1 PE=1 SV=2	3	11.178
SERPINA1	Alpha-1-antitrypsin OS=Homo sapiens OX=9606 GN=SERPINA1 PE=1 SV=3	3	11.13
GNAI2	Guanine nucleotide-binding protein G(i) subunit alpha-2 OS=Homo sapiens OX=9606 GN=GNAI2 PE=1 SV=3	2	17.885
